# Supplementary material for: Termination of non‐coding transcription in yeast relies on both an RNA Pol II CTD interaction domain and a CTD‐mimicking region in Sen1
Source: EMBO J. 2020 Feb 28;39(7):e101548. doi: 10.15252/embj.2019101548 (PMC7110113; doi:10.15252/embj.2019101548)
Supplement: Supplementary file 1 — Appendix [file EMBJ-39-e101548-s001.pdf]

Termination of non-coding transcription in yeast relies on both an RNA Pol II CTD interaction domain and a CTD-mimicking region in Sen1

Han, Z; Jasnovidova, O; Haidara, N.; Tudek, A.; Kubicek, K; Libri, D; Stefl, R and O. Porrua.

List of supplementary material:

-Appendix Figures S1-S6.

-Appendix Tables S1-S6.

-Supplementary references.

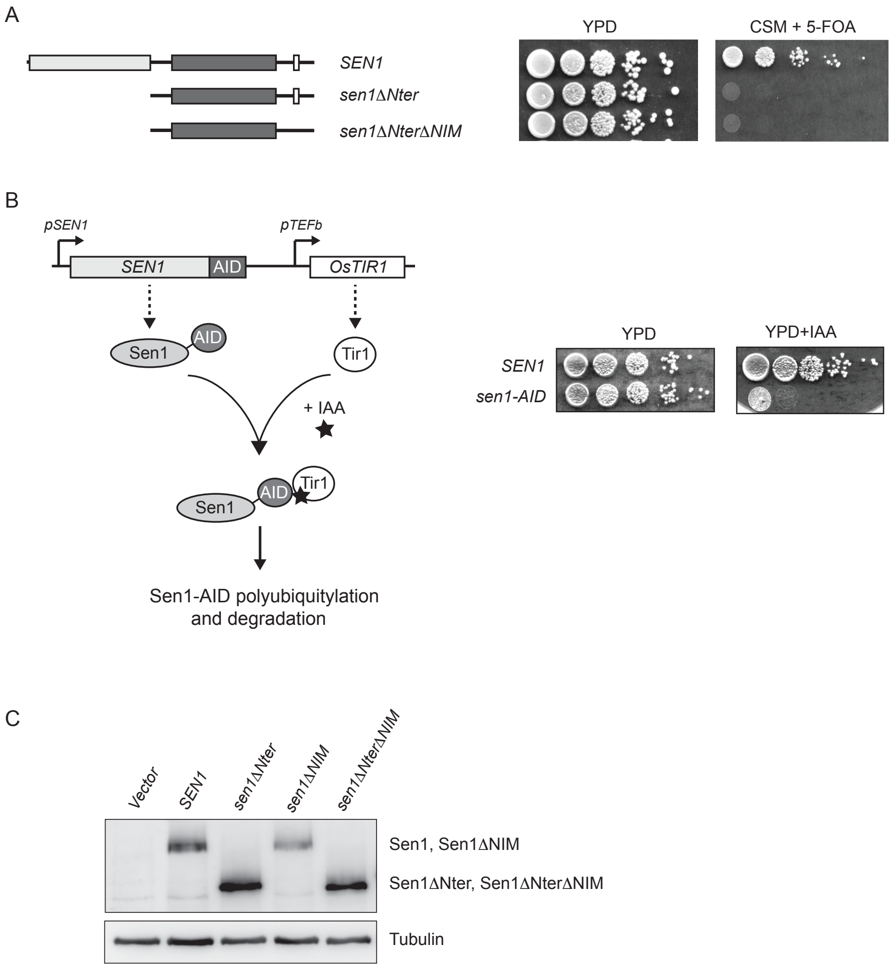

Appendix Figure S1 (related to figure 3): A) Growth tests performed as in figure 3A but using strains harbouring the TAP-tagged versions of *SEN1* indicated on the left at the endogenous locus in the presence of a *URA3* plasmid (pFL38) expressing *SEN1*. **B**) Description of the degron system employed in this study to deplete Sen1. The *SEN1* endogenous locus is modified by inserting the sequence of an AID (auxin inducible degron) tag followed by a cassette for the expression of *Oryza sativa TIR1* (*OsTIR1*), an ubiquitin ligase. In the presence of auxin hormones such as indole-3-acetic acid (IAA), Tir1 recognizes the AID-tag and promotes polyubiquitylation and subsequent degradation of Sen1-AID by the proteasome. A growth test illustrating the inhibition of cell growth upon depletion of Sen1 is shown on the right. **C**) Western blot analysis of HA-tagged versions of Sen1 expressed from a centromeric plasmid in a Sen1-AID strain upon depletion of the chromosomally-encoded copy of Sen1 as in figure 3B. Tubulin is detected as a loading control.

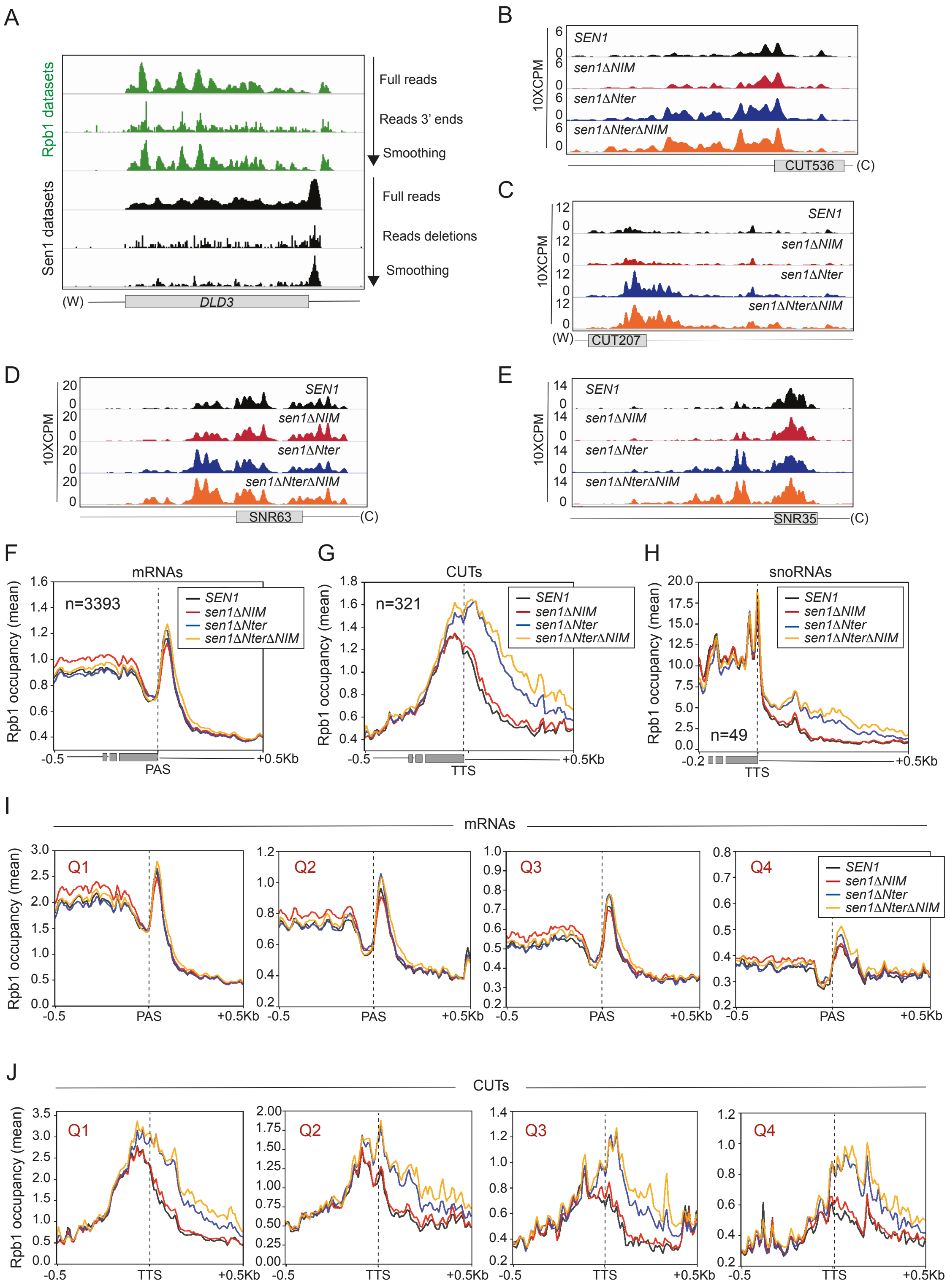

Appendix Figure S2 (related to figure 3): A) Screenshot of a particular mRNA to illustrate the conversions operated on RNAPII (Rpb1) and Sen1 CRAC datasets to increase resolution. **B**) and **C**) Additional examples of CUTs exhibiting transcription termination defects upon deletion of the Nter that are exacerbated in the double *ΔNterΔNIM* mutant. **D**) and **E**) Additional examples of snoRNAs showing transcription termination defects in Sen1 mutants. **F-H)**

Metagene analysis of the RNAPII distribution at the indicated classes of RNA in the presence of different Sen1 variants. Values on the y axis correspond to the mean coverage. Similar metagene analyses performed on quartiles for mRNAs (**I**) and CUTs (**J**), where Q1 represents the 25% of RNAs of the indicated class exhibiting the highest RNAPII occupancy in the wt.

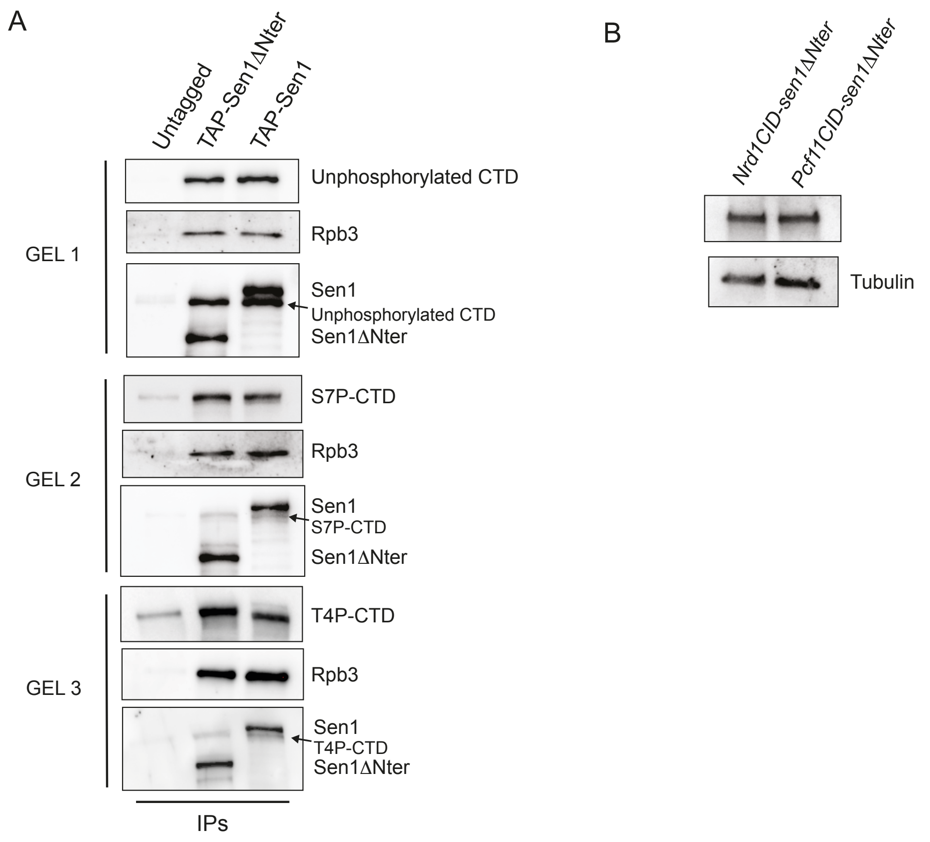

Appendix Figure S3 (related to figure 5): A) Deletion of the Sen1 N-terminal domain does not affect the interaction of Sen1 with the S7P-CTD or with the unphosphorylated CTD. CoIP experiments using TAP-Sen1 as the bait. Sen1 proteins were expressed from pGAL in the presence of galactose. The Rpb3 subunit of RNAPII is detected as a control. Protein extracts were treated with RNaseA before immunoprecipitation. Samples correspond to the same experiment and were loaded in three different gels run. **B)** Western blot analysis of HA-tagged versions of the chimeric Sen1 proteins indicated from a centromeric plasmid in a Sen1-AID strain upon depletion of the chromosomally-encoded copy of Sen1 as in figure 3B. Tubulin is detected as a loading control.

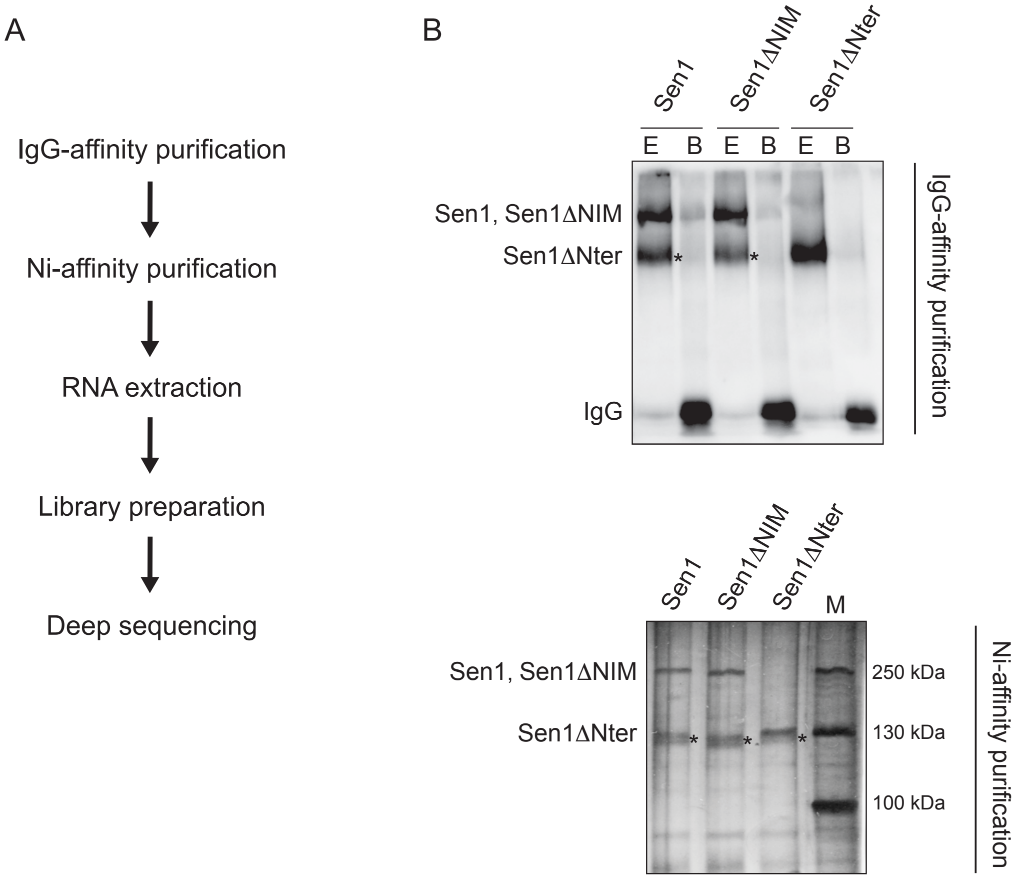

Appendix Figure S4 (related to figure 6): A) Schematic of the main steps in a CRAC experiment. B) Results of the two purification steps of the different HTP (His_6-TEV-ProteinA)-tagged Sen1 variants in a typical CRAC experiment. Representative gels corresponding to one out of two independent biological replicates. Top: western blot showing results of the IgG-affinity purification step. E, eluates after cleavage of the protein A moiety using the TEV protease. B, protein remaining associated with IgG beads. Proteins were revealed using the PAP antibody. Bottom: silver-stained denaturing gel showing the results of the Ni-affinity purification step. M, molecular-weight marker. An asterisk indicates Sen1 proteolytic fragments of size similar to that of Sen1ΔNter that we typically obtain in all Sen1 purifications.

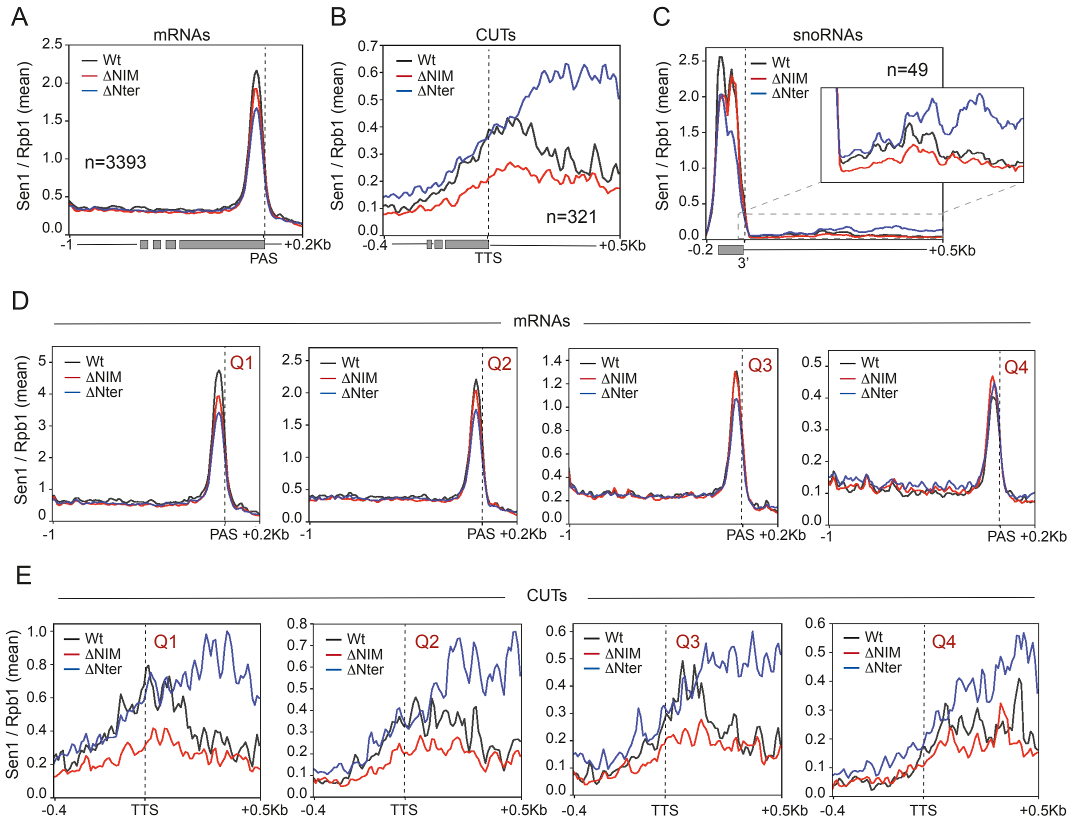

Appendix Figure S5 (related to figure 6): A-C) Metagene analysis of the normalized distribution of different Sen1 variants at the indicated classes of RNA. Values on the y axis correspond to the mean coverage. Similar metagene analyses performed on quartiles for mRNAs (**D**) and CUTs (**E**), where Q1 represents the 25% of RNAs of the indicated class exhibiting the highest wt Sen1 occupancy.

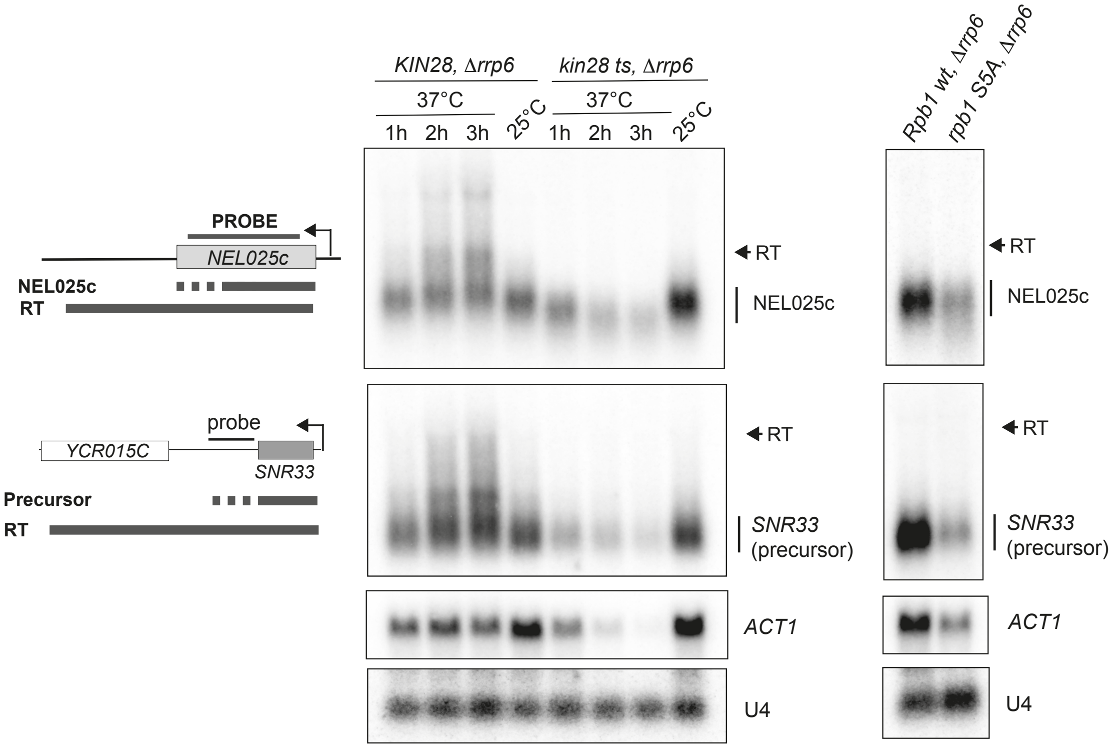

Appendix Figure S6: Analysis of the role of S5 phosphorylation for NNS-dependent transcription termination. Northern blot analyses of two well-characterized NNS-targets in either a *kin28* thermosensitive (ts) mutant (left) or a Rpb1-anchor away strain carrying a plasmid expressing either the wt or S5A versions of *RPB1* (right). Kin28 was inactivated by incubation at 37°C for the indicated times. Note that growth at 37°C induces mild termination defects in the control strain due to the presence of the *Δrrp6* mutation, which also confers a thermosensitive phenotype. The Rpb1-anchor away strains were treated for 2h with rapamycin to induce the nuclear depletion of the chromosomally-encoded Rpb1. The intermediate phenotype of this strain compared to the *kin28 ts* suggests incomplete depletion of the wt version of Rpb1 during the timeframe of the experiment. The expected RNA species resulting from inefficient termination (RT for readthrough species) are indicated. The *ACT1* mRNA is detected to provide a readout of the general decrease in transcription provoked by the inhibition of S5 phosphorylation. U4 RNA is used as a loading control. Representative gels of one out of two independent experiments. Probes used for RNA detection are described in table S14.

Appendix Table S1: Equilibrium binding of Nrd1 CID to Sen1 and Trf4 NIMs monitored by fluorescence anisotropy.

	Sen1 NIM			Trf4 NIM		
	Kd (µM)			Kd (µM)		
**wt**	1.2	±	0.02	0.9	±	0.02
**L20D**	16.9	±	1.5	13.4	±	1.2
**K21D**	8.0	±	0.8	6.1	±	0.4
**S25D**	76.1	±	14.2	53.9	±	8.2
**R28D**	78.2	±	11.5	63.4	±	4.0
**I130A**	7.6	±	0.3	4.5	±	0.2
**I130K**	36.7	±	4.6	14.1	±	1.2
**I130N**	8.9	±	0.5	6.1	±	0.3
**M126A**	1.4	±	0.07	1.1	±	0.02
**R133A**	1.8	±	0.08	1.4	±	0.02
**R133D**	2.0	±	0.1	1.2	±	0.05
**R133G**	1.6	±	0.04	1.3	±	0.04

Appendix Table S2: NMR and refinement statistics for the Nrd1 CID–Sen1 NIM complex

	Nrd1 CID-Sen1 NIM complex
**NMR distance & dihedral constraints**	
Distance restraints	
Total NOEs	2499
Intra-residue	654
Inter-residue	1845
Short	1273
Medium	645
Long	581
Hydrogen bonds	100
Intermolecular distance restraints	65
Total dihedral angle restraints[a]	222
**Structure statistics**[b]	
Violations (mean and s.d.)	
Number of distance restraint violations > 0.5 Å	0.05 ± 0.22
Number of dihedral angle restraint violations > 15º	1.1 ± 1.2
Max. dihedral angle restraint violation (º)	29.03 ± 27.63
Max. distance constraint violation (Å)	0.027 ± 0.12
Deviations from idealized geometry[b]	
Bond lengths (Å)	0.0037 ± 0.00008
Bond angles (º)	1.673 ± 0.013
Average pairwise r.m.s.d (Å)[b]	
Nrd1 CID (6-82,91-135,143-147,149-153)	
Heavy atoms	1.1
Backbone atoms	0.7
Sen1 NIM (2052-2063)	
Heavy atoms	1.9
Backbone atoms	1.3
Complex	
All complex heavy atoms	2.5
All complex backbone atoms	2.0
Ramachandran plot statistics[c]	
Residues in most favoured regions (%)	96.7
Residues in allowed regions (%)	3.2
Residues in generously allowed regions (%)	0.1
Residues in disallowed regions (%)	0.0

[a] α-helical dihedral angle restraints imposed for the backbone based on the CSI.
[b] Calculated for an ensemble of the 20 lowest energy structures.
[c] Based on Procheck analysis (Laskowski et al., 1996).

Appendix Table S3: Antibodies used in this work.

Name	Reference/provider	Working dilution	Use
Anti-HA F7	Sc-7392/ Santa Cruz	1:1000	Sen1-HA detection in figures 1A, 1C-D, 5A
Anti-HA 12CA5	11 666 606 001/ Sigma	2.5 ug/reaction	Sen1 coIP in figure 1D
Anti-CBP, calmodulin binding protein	07-482/ Millipore	1:1000	Nrd-CBP detection in figure 1A, Sen1-CBP detection in figure 5B
Anti-Air2	S. Vanacova	1:1000	Air2 detection in figure 1A
Anti-Rpb1 y80	Santa Cruz (not available any more)	1:1000	Rpb1 detection in figure 5A, 5B
Anti-phospho-CTD Ser5 clone 3E8	04-1572/ Millipore	1:1000	Detection of S5P-CTD of RNAP II in figure 5B
Anti-phospho-CTD Ser2 clone 3E10	04-1571/ Millipore	1:1000	Detection of S2P-CTD of RNAP II in figure 5B
Anti-phospho-CTD Ser7 clone 4E12	04-1570/ Millipore	1:1000	Detection of S7P-CTD of RNAP II in figure S3
Anti-phospho-CTD Thr4 clone	61362/Active motif	1:500	Detection of T4P-CTD of RNAP II in Appendix Figure S3
Anti-CTD 8WG16	Ab817/abcam	1:1000	Detection of the unphosphorylated CTD preferentially.
PAP, peroxidase anti-peroxidase	P1291-1ML/ Sigma	1:3000	Used for Protein A detection on TAP tag.
Anti-Nrd1	Covalab	1:3000	Nrd1 detection in figures 1 and 5
Anti-Nab3	Covalab	1:3000	Nab3 detection in figures 1 and 5
Anti-His	H1029,Sigma	1:2000	His_6-GST-Sen1 Cter and His_6-GST in figure 5E

Appendix Table S4: Yeast strains used in this work.

Number	Name	Genotype	Source
DLY17	W303	*ura3-1, ade2-1, his3-11,5, trp1-1, leu2-3,112, can1-100*	(Thomas and Rothstein, 1989)
DLY467	KIN28	*kin28::URA, [pSF19-KIN28, CEN, TRP]*	(Cismowski et al., 1995)
DLY468	kin28 ts16	*kin28::URA, [pSF19-kin28-ts16 CEN, TRP]*	(Cismowski et al., 1995)
DLY671	BMA	*as W303, Δtrp1*	F. Lacroute
DLY814	*Δrrp6*	*as W303, rrp6::KAN*	(Porrua et al., 2012)
DLY1657	*sen1-HA*	*as BMA, Sen1::HA::KAN*	(Tudek et al, 2014
DLY2014	*nrd1 ΔCID-TAP, sen1-HA*	*as BMA, nrd1ΔCID (Δ6-150)::TAP::HIS3*	Tudek et al, 2014
DLY2769	*sen1ΔNIM*	*as BMA, sen1ΔNIM (Δ2052-2063)*	This work
DLY2982	*sen1ΔNIM-HA*	*as BMA, sen1ΔNIM (Δ2052-2063)::HA::KAN*	This work
DLY1737	*nrd1-TAP, sen1-HA*	*as BMA, NRD1::TAP::HIS5 Sen1::HA::KAN*	(Tudek et al, 2014)
DLY2622	*nrd1-TAP, sen1ΔNIM-HA*	*as BMA, NRD1::TAP::HIS5, sen1ΔNIM (Δ2052-2063)::HA::KAN*	This work
DLY2657	*sen1ΔCter-HA*	*as BMA, sen1ΔCter (Δ1930-2231)::HA::KAN*	This work
DLY2658	*nrd1-TAP, sen1ΔCter-HA*	*as BMA, NRD1::TAP::HIS5, sen1ΔCter (Δ1930-2231)::HA::KAN*	This work
DLY2695	*sen1ΔCter-HA, Δrrp6*	*as BMA, sen1ΔCter (Δ1930-2231)::HA::KAN, rrp6::URA*	This work
DLY2694	*sen1-HA, Δrrp6*	*as BMA, sen1::HA::KAN, rrp6::URA*	This work
DLY2770	*sen1ΔNIM, Δrrp6*	*as BMA, sen1ΔNIM, rrp6::KAN*	This work
DLY2767	*Δsen1*/pFL38-*SEN1*	*as BMA, sen1::KAN, harbouring plasmid pFL38-SEN1*	This work
DLY1656	*P_{GAL1}- TAP-SEN1*	*as BMA, TRP1::Pgal::TAP::SEN1*	(Porrua et al., 2012)
DLY2778	*P_{GAL1}- TAP-sen1ΔNIM*	*as BMA, TRP1::Pgal::TAP::sen1ΔNIM (Δ2052-2063)*	This work
DLY2692	*P_{GAL1}- TAP-sen1ΔNter*	*as BMA, TRP1::Pgal::TAP::sen1ΔNter (Δ1-975)*	This work
DLY2779	*P_{GAL1}- TAP-sen1ΔNter ΔNIM*	*as BMA, TRP1::Pgal::TAP::sen1ΔNter (Δ1-975) ΔNIM (Δ2052-2063)*	This work
DLY2060	*P_{GAL1}- TAP-SEN1, Δrrp6*	*as BMA, TRP1::Pgal::TAP::SEN1, rrp6::KAN*	(Porrua et al., 2012)
DLY2780	*P_{GAL1}- TAP-sen1ΔNIM, Δrrp6*	*as BMA, TRP1::Pgal::TAP::sen1ΔNIM (Δ2052-2063), rrp6::KAN*	This work
DLY2698	*P_{GAL1}- TAP-sen1ΔNter,Δrrp6*	*as BMA, TRP1::Pgal::TAP::sen1ΔNter (Δ1-975), rrp6::KAN*	This work

DLY2781	*P_{GAL1}- TAP-sen1ΔNterΔNIM, Δrrp6*	*as BMA, TRP1::Pgal::TAP::sen1ΔNter (Δ1-975) ΔNIM (Δ2052-2063), rrp6::KAN*	This work
DLY2782	*sen1-AID*	*as BMA,SEN1-AID::KAN::OsTIR1*	This work
DLY2788	*sen1-AID, Δrrp6*	*as BMA,SEN1-AID::KAN::OsTIR1, rrp6::URA*	This work
DLY2724	*TAP-sen1/*pFL38-*SEN1*	*as BMA, TAP::SEN1, harbouring plasmid pFL38-SEN1*	This work
DLY2725	*TAP- sen1ΔNter /*pFL38-*SEN1*	*TAP::sen1ΔNter (Δ1-975), harbouring plasmid pFL38-SEN1*	This work
DLY2726	*TAP- sen1ΔNterΔNIM /*pFL38-*SEN1*	*as BMA, TAP::sen1ΔNter (Δ1-975) ΔNIM (Δ2052-2063), harbouring plasmid pFL38-SEN1*	This work
DLY3152	*P_{GAL1}- TAP-SEN1, Δnrd1/ pRS316-NRD1*	*as BMA, TRP1::Pgal::TAP::SEN1, nrd1::KAN; harbouring plasmid pRS316-NRD1*	This work
DLY3187	*P_{GAL1}- TAP-SEN1, Δnab3/ pFL38-Nab3*	*as BMA, TRP1::Pgal::TAP::SEN1, nab3::KAN; harbouring plasmid pFL38-NAB3*	This work
DLY3081	*rpb3-FLAG*	*as BMA, RPB3::3xFLAG::natMX6*	This work
DLY3151	*rpb3-FLAG, sen1-AID*	*as BMA, SEN1-AID:KAN::OsTIR1, RPB3::3xFLAG::natMX6*	This work
DLY3361	*sen1-AID, rpb1-HTP*	*as BMA, Sen1::AID, KAN::OsTIR1, rpb1-HTP::URA*	This work
yFR1499	Rpb1 anchor away + Rpb1-CTD wt-3xFLAG	*ade2-1, trp1-1, can1-100, leu2-3,112, his3-11,15, ura3, GAL, psi+, tor1-1, fpr1Δ::NAT, RPL13A-2xFKBP12::TRP1, RPB1-FRB::KanMX6, [pFR482 (CEN, HIS3, RPB1-CTDWT-3xFLAG)]*	(Collin et al., 2019)
yFR1503	Rpb1 anchor away + Rpb1-CTD S5A-3xFLAG	*ade2-1, trp1-1, can1-100, leu2-3,112, his3-11,15, ura3, GAL, psi+, tor1-1, fpr1Δ::NAT, RPL13A-2xFKBP12::TRP1, RPB1-FRB::KanMX6, [pFR490 (CEN, HIS3, rpb1-CTDS5A-3xFLAG)]*	(Collin et al., 2019)
DLY3423	*Δrrp6,* Rpb1 anchor away + Rpb1-CTD wt-3xFLAG	*as yFR1499, rrp6::URA*	This work
DLY3424	*Δrrp6,* Rpb1 anchor away + Rpb1-CTD S5A-3xFLAG	*as* yFR1503, *rrp6::URA*	This work
DLY3417	*KIN28, Δrrp6*	*as YDL467, rrp6::KAN*	This work
DLY3419	*kin28 ts16, Δrrp6*	*as YDL468, rrp6::KAN*	This work

Appendix Table S5: plasmids used in this work.

**Name**	**Description**	Source
pBS1761	Ap^r, *ori*ColE1; plasmid bearing cassette for N-terminal tagging with PGAL1-TAP	(Finoux and Séraphin, 2006)
pDL708 (pU6H3HA)	Ap^r, *ori*ColE1; plasmid bearing cassette for C-terminal tagging with *HA*	(Finoux and Séraphin, 2006)
pDL772	Ap^r; *ori*ColE1; derivative of pFL38 (URA) bearing yeast *SEN1*	F. Lacroute
pDL693	Ap^r; *ori*ColE1; derivative of pFL39 bearing yeast *SEN1*	F. Lacroute
pDL703	Ap^r; *ori*ColE1; derivative of pFL39 bearing *sen1ΔNIM*	This work
pETM30	Kan^r; oriColE1; vector for overexpression of proteins from the T7 promoter	R. Stefl
pDL834	Ap^r; *ori*ColE1; derivative of pFL39 bearing *sen1ΔNter*	This work
pDL835	Ap^r; *ori*ColE1; derivative of pFL39 bearing *sen1ΔNterΔNIM*	This work
pDL846	Kan^r; oriColE1; derivative of pETM30 carrying the C-terminal domain of Sen1 (aa 1931-2231) under the control of the T7 promoter	This work
pDL848	Kan^r; oriColE1; derivative of pETM30 carrying the ΔNIM version of the C-terminal domain of Sen1 (aa 1931-2231) under the control of the T7 promoter	This work
pDL856	Ap^r; *ori*ColE1; derivative of pFL39 bearing yeast se*n1-HA*	This work
pDL858	Ap^r; *ori*ColE1; derivative of pFL39 bearing *sen1ΔNIM-HA*	This work
pDL857	Ap^r; *ori*ColE1; derivative of pFL39 bearing *sen1ΔNter-HA*	This work
pDL859	Ap^r; *ori*ColE1; derivative of pFL39 bearing *sen1ΔNterΔNIM-HA*	This work
pDL876	Ap^r; *ori*ColE1; derivative of pFL39 bearing the chimeric *nrd1CID* (aa 1-153) - *sen1ΔNter*	This work
pDL887	Ap^r; *ori*ColE1; derivative of pFL39 bearing the chimeric *nrd1CID* (aa 1-153) - *sen1ΔNter-HA*	This work
pDL965	Ap^r; *ori*ColE1; derivative of pFL39 bearing the chimeric *pcf11CID* (aa 1-137) - *sen1ΔNter*	This work
pDL966	Ap^r; *ori*ColE1; derivative of pFL39 bearing yeast se*n1-HTP*	This work
pDL967	Ap^r; *ori*ColE1; derivative of pFL39 bearing yeast *sen1ΔNIM -HTP*	This work
pDL968	Ap^r; *ori*ColE1; derivative of pFL39 bearing yeast *sen1ΔNter -HTP*	This work
pDL991	Ap^r; *ori*ColE1; derivative of pFL39 bearing the chimeric *pcf11CID* (aa 1-137) - *sen1ΔNter-HA*	This work
pRS_NC	Ap^r; oriColE1; plasmid bearing CID-His6 under the control of the T7 promoter	(Kubicek et al., 2012)
pRS_NC_L20D	Kan^r; oriColE1; plasmid bearing CID(L20D)-His6 under the control of the T7 promoter	(Vasiljeva et al., 2008)
pRS_NC_K21D	Kan^r; oriColE1; plasmid bearing CID(K21D)-His6 under the control of the T7 promoter	(Vasiljeva et al., 2008)
pRS_NC_S25R	Ap^r; oriColE1; plasmid bearing CID(S25R)-His6 under the control of the T7 promoter	(Kubicek et al., 2012)
pRS_NC_R28D	Ap^r; oriColE1; plasmid bearing CID(R28D)-His6 under the control of the T7 promoter	(Kubicek et al., 2012)
pRS_NC_I130A	Ap^r; oriColE1; plasmid bearing CID(I130A)-His6 under the control of the T7 promoter	(Tudek et al., 2014)
pRS_NC_I130K	Ap^r; oriColE1; plasmid bearing CID(I130K)-His6 under the control of the T7 promoter	(Tudek et al., 2014)
pRS_NC_I130N	Ap^r; oriColE1; plasmid bearing CID(I130N)-His6 under the control of the T7 promoter	(Tudek et al., 2014)
pRS_NC_R133A	Ap^r; oriColE1; plasmid bearing CID(R133A)-His6 under the control of the T7 promoter	(Tudek et al., 2014)
pRS_NC_R133G	Ap^r; oriColE1; plasmid bearing CID(R133G)-His6 under the control of the T7 promoter	This work
pRS_NC_R133D	Ap^r; oriColE1; plasmid bearing CID(R133D)-His6 under the control of the T7 promoter	This work

Appendix Table S6: List of oligonucleotides used in this work.

Name	Sequence (5'-3')	Information/use
DL1119	AAGTGACGAAGTTCATGCTA	Forward oligo to generate by PCR a probe to detect snR13 read-through region.
DL1367	GGCCCAACAGTATATTCATATCC	Reverse oligo to generate by PCR a probe to detect snR13 read-through region.
DL474	GCAAAGATCTGTATGAAAGG	Forward oligo to generate by PCR a probe to detect NEL025C.
DL480	ATCTGACCAGGTCAAGCTAC	Reverse oligo to generate by PCR a probe to detect NEL025C.
DL2505	GTGTGTGGACAATCGATTTGC	Forward oligo to generate by PCR a probe to detect snR33 3'precursor and read-through region
DL2506	GCATTGGCTCGATTGTCAAC	Reverse oligo to generate by PCR a probe to detect snR33 3'precursor and read-through region
DL1154	CCTATAACAACAACAACATG	Forward oligo to generate by PCR a probe to detect snR47 RNA.
DL1157	ATAGCCATTAGTAAGTACGC	Reverse oligo to generate by PCR a probe to detect snR47 RNA.
DL2627	ATTCAAAAGCGAACACCGAATTGAC CATGAGGAGACGGTCTGGTTTAT	Reverse oligo used as oligo probe to detect U4 snRNA
DL377	ATGTTCCCAGGTATTGCCGA	Forward oligo to generate by PCR a probe to detect *ACT1* RNA.
DL378	ACACTTGTGGTGAACGATAG	Reverse oligo to generate by PCR a probe to detect *ACT1* RNA.

Supplementary references:

Cismowski, M.J., Laff, G.M., Solomon, M.J., and Reed, S.I. (1995). KIN28 encodes a C-terminal domain kinase that controls mRNA transcription in Saccharomyces cerevisiae but lacks cyclin-dependent kinase-activating kinase (CAK) activity. Mol. Cell. Biol. *15*, 2983–2992.

Finoux, A.-L., and Séraphin, B. (2006). In vivo targeting of the yeast Pop2 deadenylase subunit to reporter transcripts induces their rapid degradation and generates new decay intermediates. J. Biol. Chem. *281*, 25940–25947.

Laskowski, R.A., Rullmannn, J.A., MacArthur, M.W., Kaptein, R., and Thornton, J.M. (1996). AQUA and PROCHECK-NMR: programs for checking the quality of protein structures solved by NMR. J. Biomol. NMR *8*, 477–486.

Thomas, B.J., and Rothstein, R. (1989). The genetic control of direct-repeat recombination in Saccharomyces: the effect of rad52 and rad1 on mitotic recombination at GAL10, a transcriptionally regulated gene. Genetics *123*, 725–738.
