# Supplementary material for: Termination of non‐coding transcription in yeast relies on both an RNA Pol II CTD interaction domain and a CTD‐mimicking region in Sen1
Source: EMBO J. 2020 Feb 28;39(7):e101548. doi: 10.15252/embj.2019101548 (PMC7110113; doi:10.15252/embj.2019101548)
Supplement: Supplementary file 2 — Expanded View Figures PDF [file EMBJ-39-e101548-s002.pdf]

Expanded View Figures

Figure EV1. The Sen1 NIM is conserved in close yeast species.
Saccharomyces cerevisiae Sen1 protein sequence was submitted to blastp excluding the genus *Saccharomyces* from the search. The ten most conserved protein sequences together with Sen1 orthologues from *Homo sapiens* (SETX) and *Schizosaccharomyces pombe* were aligned to *S. cerevisiae* Sen1 using clustal omega. Visualization of the alignment and calculation of the consensus sequences ware performed with Jalview (Waterhouse *et al*, 2009). Amino acids are coloured according to clustal colour scheme. The protein identifiers for the proteins used in the alignment are the following: Q7Z333 (*H. sapiens*), Q92355 (*S. pombe*), XP_001644478.1 (*Vanderwaltozyma polyspora*), SMN21961.1 (*Kazachstania saulgeensis*), SCV99407.1 (*Lachancea fermentati*), CCK69072.1 (*Kazachstania naganishii*), XP_004181671.1 (*Tetrapisispora blattae*), XP_003955130.1 (*Kazachstania Africana*), XP_003672383.1 (*Naumovozyma dairenensis*), Q00416 (*S. cerevisiae*), XP_003680903.1 (*Torulaspora delbrueckii*), CDF91445.1 (*Zygosaccharomyces bailii*) and GAV52597.1 (*Zygosaccharomyces rouxii*). The NIM is indicated by a red box.

© 2020 The Authors

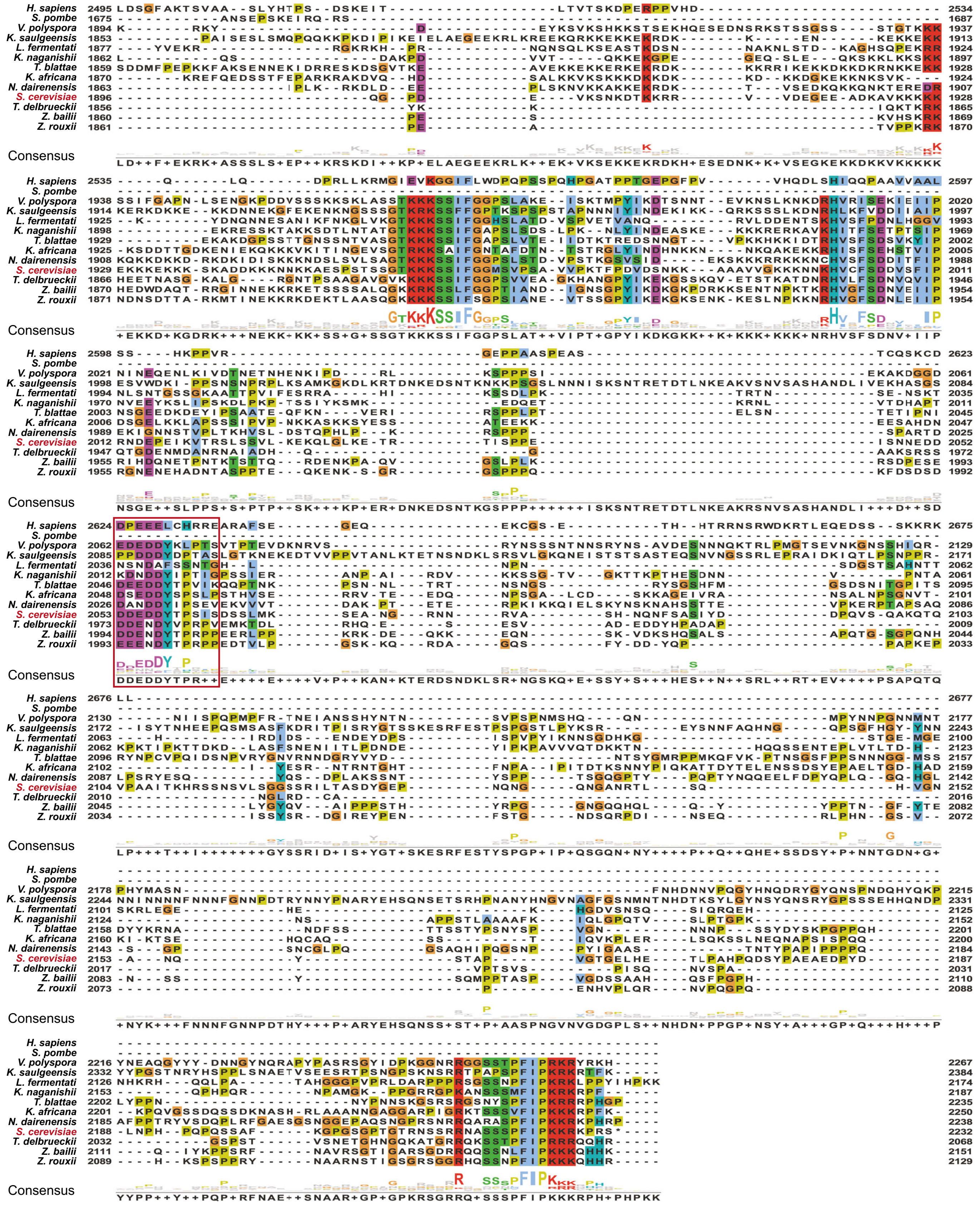

Figure EV1.

© 2020 The Authors

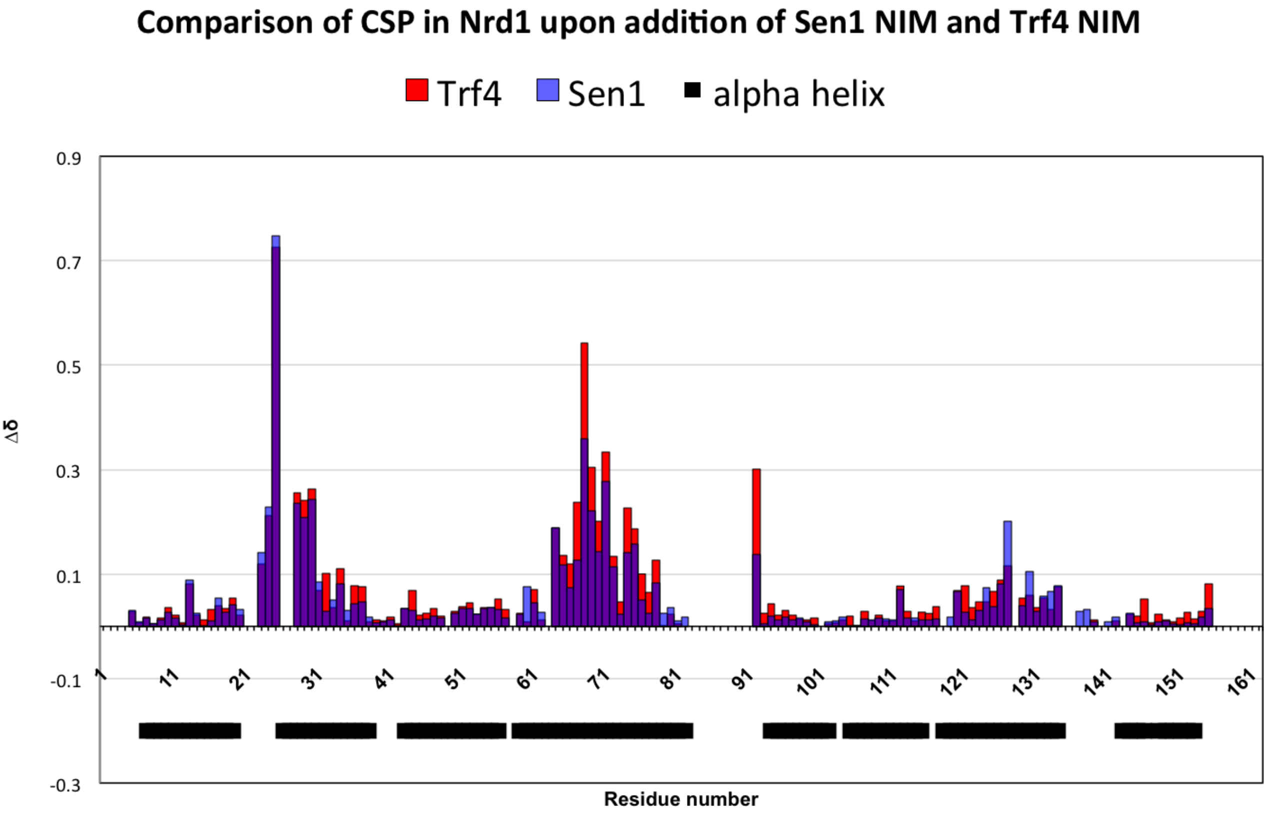

Figure EV2. Quantification of chemical shift perturbations (CSP) of Nrd1 CID upon binding to the Trf4 NIM (in red) and Sen1 NIM peptides (in blue).
The combined chemical shift perturbations are plotted versus the amino acid residue number. The black boxes indicate α-helices of Nrd1 CID.

Figure EV3. The interaction of Sen1 with Nrd1 and Nab3 is not essential for non-coding transcription termination (related to Fig 3).

A Growth tests of the *sen1ΔNIM* mutant in either a wt or a Δ*rrp6* background.

B–D Metagene analyses of RNAseq experiments performed in a Δ*rrp6* background in the presence of the wt or the Δ*NIM* version of Sen1. The profile corresponds to the median coverage (reads per 10^7 reads mapping at each genomic position) from 0.5 kb upstream to 0.5 kb downstream of the annotated transcription termination site (TTS) of protein-coding genes (B) and CUTs (D) or the 3′ end of the mature snoRNAs (C). Experiments were performed in biological duplicates.

E Deletion of Sen1 C-terminal domain completely abolishes the interaction of Sen1 with Nrd1. Top: scheme of proteins analysed in these experiments. Bottom: CoIP assays using Nrd1-TAP as the bait. Representative gel of one out of two independent experiments. Antibodies used for protein detection are detailed in Appendix Table S3.

F Deletion of Sen1 Cter provokes minor transcription termination defects at typical NNS-dependent non-coding genes. Northern blot assays performed in a Δ*rrp6* background. Results correspond to one out of two independent biological replicates. The *CAR2* and *PHO5* RNAs are detected as a loading controls. Probes used for RNA detection are described in Appendix Table S6.

© 2020 The Authors

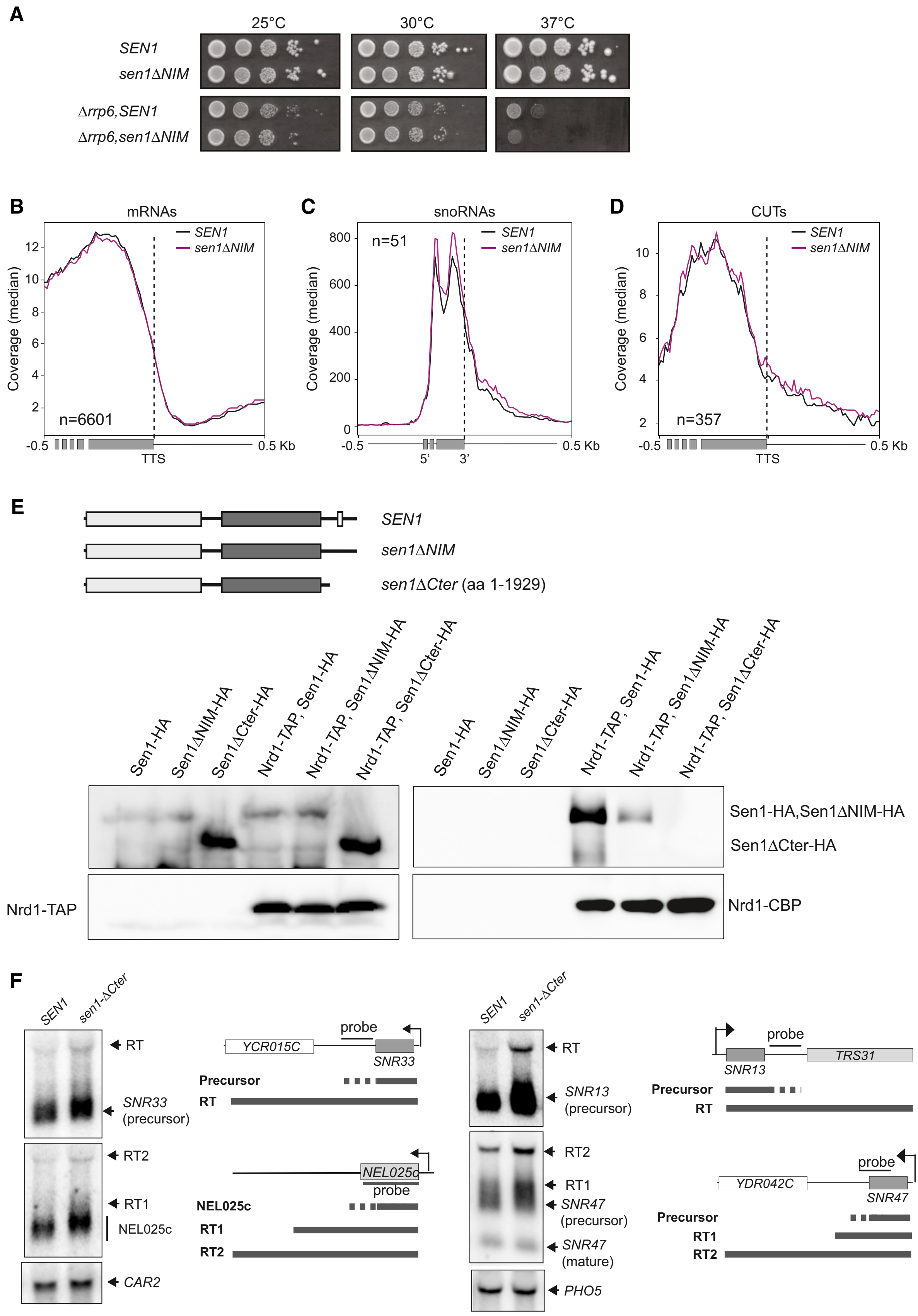

Figure EV3.

 © 2020 The Authors

Figure EV4. Quantification of transcription termination defects detected in the different Sen1 mutants at individual ncRNAs (related to Fig 4).

A Plot representing the RT index of the indicated mutants relative to the wt version of Sen1 for each CUT. RT indexes were calculated as in Fig 4. CUTs are ranked in the decreasing order according to corresponding value in *sen1ΔNter*. Data from *sen1ΔNIM* and *sen1ΔNterΔNIM* are shown by vertical bars while data from *sen1ΔNter* are represented by a line.

B–D Heatmaps representing the log2 of the fold change (FC) of the RNAPII signal in the indicated mutants relative to the wt at CUTs.

E Graph representing the RT index, calculated as in Fig 4, of the indicated mutants relative to the wt for each snoRNA analysed.

F–H Heatmaps showing the log2 FC of the signal in the indicated mutants relative to the wt at snoRNAs.

© 2020 The Authors

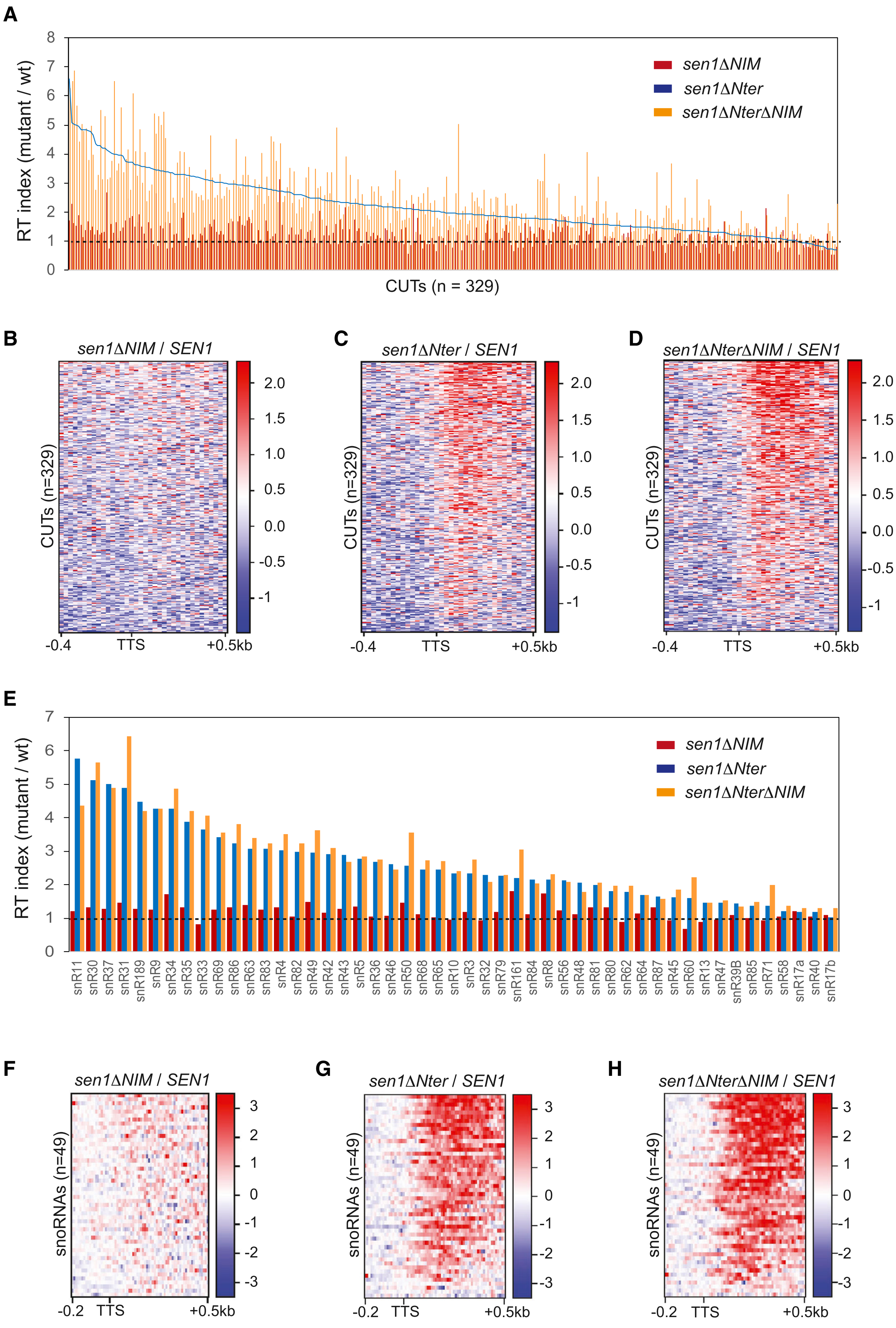

Figure EV4.

© 2020 The Authors

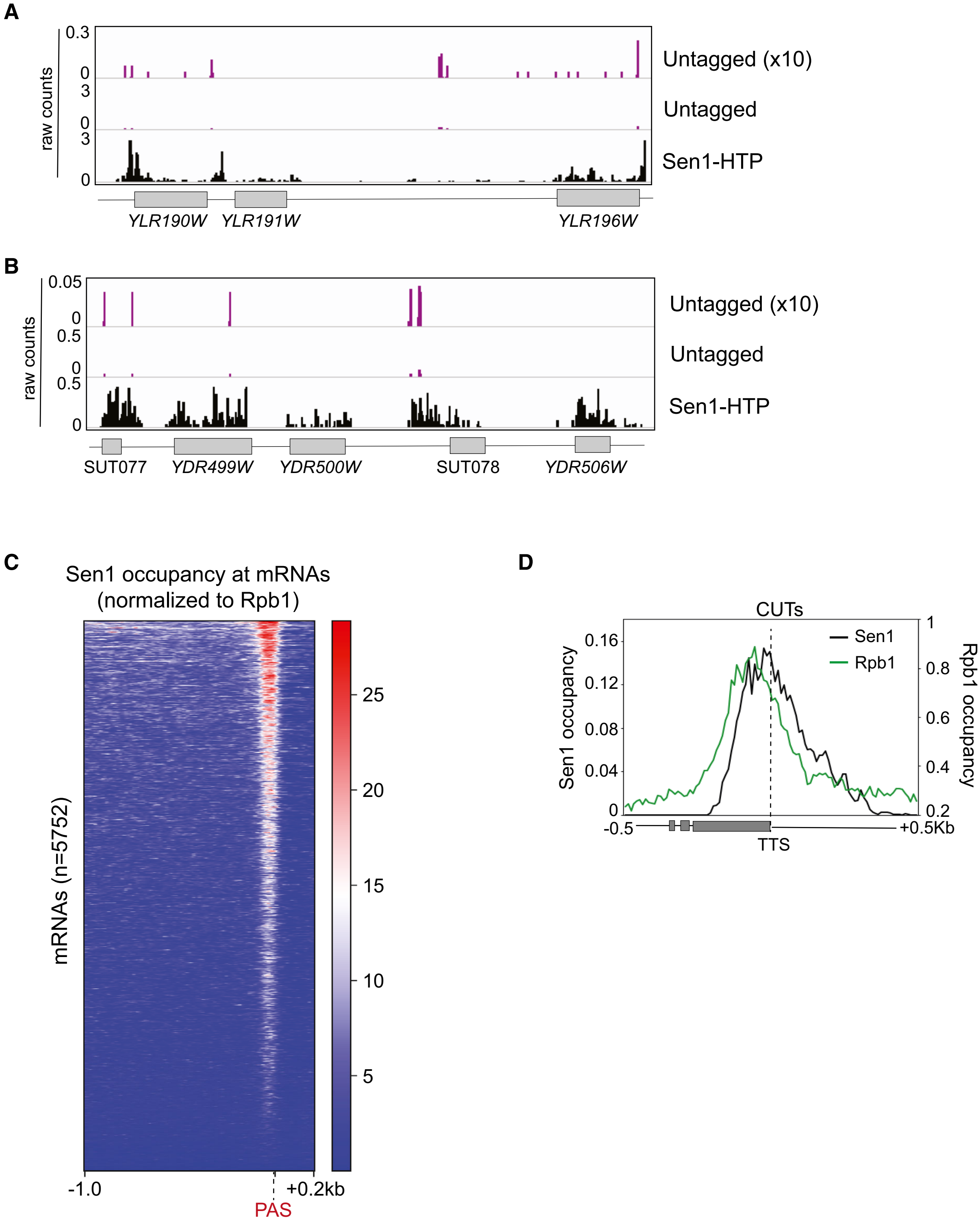

Figure EV5. Distribution of Sen1 signal at different types of RNAs (related to Fig 5).

A, B Screenshots of particular genomic regions to illustrate the differences in the signals obtained with the untagged control, relative to a strain expressing the HTP-tagged version of Sen1 allowing its purification (see Methods for details). The signal of the control is multiplied by 10 to allow a better comparison with that of Sen1-HTP. Values correspond to the data before normalization to total number of deletions. The data from one of the biological replicates of Sen1 wt is shown as Sen1-HTP.

C Heatmap visualization of the distribution of Sen1 wt at mRNAs.

D Metagene analysis of the distribution of the different Sen1 variants at CUTs as in Fig 6J but using the TTS as reference point.
